# Designing for Autonomous Motivation: Qualitative Interview Study on Pre-Enrollment Preferences of Survivors of Cancer for Digital Health Behavior Change Programs

**DOI:** 10.2196/87514

**Published:** 2026-08-10

**Authors:** Monisola Jayeoba, Rachel Sohn, Abigail Louise Weiss, Anja Stanic, Rana Mazzetta, Alice Pham, Laura Danielle Scanlan, Mario Garcia, Armaan Sidhu, Elizabeth Ho, Sofia F Garcia, Brian Hitsman, Siobhan M Phillips, Bonnie Spring, Maia Jacobs

**Affiliations:** 1 Department of Communication Studies, School of Communication, Northwestern University, Evanston, IL, United States; 2 Department of Computer Science, McCormick School of Science and Engineering, Northwestern University, Mudd Building, 3rd Floor, 2233 Tech Drive, Evanston, IL, 60201, United States, 1 847 491 2861; 3 Department of Preventive Medicine, Feinberg School of Medicine, Northwestern University, Chicago, IL, United States; 4 Department of Physical Medicine & Rehabilitation, Feinberg School of Medicine, Northwestern University, Chicago, IL, United States; 5 Division of Gastroenterology and Hepatology, Feinberg School of Medicine, Northwestern University, Chicago, IL, United States; 6 Department of Dermatology, Feinberg School of Medicine, Northwestern University, Chicago, IL, United States; 7 Department of Psychiatry and Behavioral Sciences, Feinberg School of Medicine, Northwestern University, Chicago, IL, United States; 8 Robert H. Lurie Comprehensive Cancer Center, Northwestern University, Chicago, IL, United States; 9 Department of Medical Social Sciences, Feinberg School of Medicine, Northwestern University, Chicago, IL, United States; 10 Department of Behavioral Science and Social Medicine, College of Medicine, Florida State University, Tallahassee, FL, United States

**Keywords:** digital health behavior change, cancer survivorship, self-determination theory, user-centered design, autonomous motivation

## Abstract

**Background:**

Despite elevated recurrence risks associated with modifiable lifestyle factors, many survivors of cancer do not adhere to health promotion recommendations. Digital health interventions hold promise for supporting behavior change, but few studies involve survivors in intervention design, potentially limiting real-world effectiveness. Understanding the preferences and support needs of survivors of cancer is crucial for developing effective digital health behavior change interventions.

**Objective:**

The aim of the study is to understand pre-enrollment perspectives of survivors of cancer on support needs and preferences regarding optimal treatment features in digital health behavior change interventions.

**Methods:**

We conducted in-depth semistructured interviews with 16 survivors of cancer who were contemplating participating in a multiple behavior change intervention pilot study. Participants were asked about their past experiences with behavior change and to envision ideal intervention features relative to their survivorship journey. This study followed an inductive thematic analysis framework, and findings were reported from a self-determination theory perspective. Six researchers were involved in the coding process to ensure analytical rigor.

**Results:**

The study identified 4 main themes about needs and preferences for digital behavior change interventions. These primarily mapped onto three psychological needs from self-determination theory: (1) competence through technology-enabled tracking, skill development, and expert accessibility; (2) autonomy through personalized, flexible program design acknowledging existing knowledge; and (3) relatedness through human-centered systems featuring health promotionists and health care team integration. A fourth subtheme emerged that highlights participants’ desire for integrated, comprehensive care systems that connected behavior change programs with broader health care ecosystems rather than functioning as stand-alone digital programs.

**Conclusions:**

This study demonstrates that survivors of cancer have an ample understanding of their motivational needs and can offer valuable guidance for intervention design when given the opportunity to articulate their preferences. Our findings address a critical gap in the literature, where survivor perspectives remain underrepresented in digital health design. By centering survivor voices in the pre-enrollment phase, this study provides actionable insights for developing interventions that support progression from externally driven participation to sustained, autonomous engagement. Overall, this work offers a roadmap for designing flexible, human-centered, and integrated digital health interventions that can better support long-term behavior change in cancer survivorship.

## Introduction

### Background

Improvements in early detection, diagnosis, and treatments have contributed to a rapidly expanding cancer survivor population [1-4], with an estimated 18 million US [5] survivors in 2022, and projections reaching 22 million by 2030 [6]. However, adult survivors of cancer who have completed curative-intent treatment are at risk of cancer recurrence, second cancers, and cardiometabolic disease associated with modifiable risk factors, including physical inactivity, smoking, and poor weight management [7-10]. Approximately 30%‐40% of cancers are associated with these modifiable lifestyle factors [11], and at least 1 in 3 survivors of cancer engage in one of the 3 prevalent risk behaviors [12]. Even though major professional organizations, including the American Cancer Society [13-18], American Society of Clinical Oncology [17,19,20], and National Cancer Center Network [21] recommend incorporating lifestyle management and health promotion into survivorship care plans [22-25], many survivors of cancer do not meet the health promotion guidelines for these modifiable risk factors [12,15,26,27]. This makes the postcurative treatment period a critical window for behavioral interventions [9,10,28-30].

Digital health interventions have become ubiquitous and hold significant promise in cancer survivorship care [31-33] for risk reduction and self-management of healthy lifestyles [34-38], including physical activity [31,39-42], smoking cessation [43,44], and weight management [45,46]. These technologically enabled interventions offer survivors of cancer continuous support between sparse clinical visits after curative treatment [47], improved health care access in rural or underserved communities [48-51], and reduced cost of care. Yet, recent research reveals a fundamental gap in how these interventions are developed [52]. A 2025 scoping review found that despite the recognized importance of perspectives of survivors of cancer, few digital health behavior change interventions meaningfully involve survivors in the research design and development [52]. Several studies have also shown that digital interventions developed without meaningful user input are more likely to experience nonadoption, abandonment, and implementation failures [53,54]. This gap represents a significant missed opportunity, as interventions developed without substantial survivors’ input may fail to address patients’ real-world motivational challenges and needs, thereby undermining long-term behavior change success [55,56]. Our study addresses this critical gap by centering the voices of survivors of cancer in the pre-enrollment phase, capturing their motivation for participation, support needs, and preferences before they enroll in a digital health behavior change program. Understanding patients’ requirements and preferences should increase the likelihood that patients’ needs will be met. That, in turn, should lead to better patient adoption and use of digital health solutions [55,57], thereby improving health outcomes [58] and avoiding clinical research waste [59].

Decades of behavioral research consistently demonstrate that motivation, more than knowledge, resources, or even initial health status, predicts who will successfully initiate and maintain health behavior change [60-63]. Autonomous motivation promotes sustained behavioral persistence, positive affect, and effective performance while also supporting psychological well-being and healthy development [61,64-66]. Since changing health behaviors is rarely inherently pleasurable, pure intrinsic motivation can be difficult to sustain in health contexts. However, by satisfying individuals’ basic psychological needs for competence, autonomy, and relatedness, extrinsic motivations can become increasingly autonomous, as individuals learn to accept personal ownership of behaviors and integrate them with their sense of self [67-69]. When these needs are met, individuals can maintain motivation even when external incentives diminish.

### This Study and Objectives

In cancer survivorship, initial participation in health behavior change programs is often driven by extrinsic motivating factors such as fear of disease recurrence or clinician recommendations. While the “teachable moment” of cancer diagnosis creates openness to change [29,70-73], these external motivators frequently diminish over time, decreasing engagement when it is most essential for sustaining behavior change. Understanding patients’ support needs and preferences for intervention design could help foster autonomous motivation within environments that fundamentally rely on extrinsic incentives. Hence, this study aims to understand the support needs and pre-enrollment preferences of survivors of cancer for digital health behavior change programs. Specifically, the study examines (1) factors influencing motivation and engagement in these programs and (2) design recommendations for supporting behavior change.

We conducted in-depth semistructured interviews with 16 survivors of cancer eligible for an upcoming first-of-its-kind, digital health intervention trial [74], asking them to envision ideal intervention features relative to their survivorship journey. Participants shared what would enhance their motivation to stay engaged with future interventions. We used a hybrid inductive-deductive thematic analysis [75,76], combining inductive coding with theoretical refinement. This 2-phase analytical process allowed us to remain grounded in participants’ experiences while leveraging theoretical constructs to deepen our interpretation. The findings offer actionable recommendations for developing flexible, human-centered, integrated digital health behavior change interventions. Adopting these approaches could help address the critical gap between evidence-based health behavior recommendations and current survivor adherence patterns.

## Methods

### Overview or Study Setting

This study was conducted at a National Cancer Institute–designated comprehensive cancer center within a large university medical network located in Chicago and its suburbs. It served as pilot research conducted preparatory to launching a novel, scalable telehealth cancer care clinical trial [74] focused on testing a telehealth-based intervention that addresses multiple behavioral risk factors at once. A key objective of this study was to capture the perspectives of survivors of cancer during pre-enrollment, when they are contemplating participation in a health behavior change program but have not yet committed to a specific intervention. This approach provides unique insights into both initial motivational drivers and what survivors believe will sustain their efforts over time, before being constrained by actual program experience. One-on-one semistructured interviews were conducted with patients who had completed active cancer treatment and were receiving postcurative survivorship care and support at the cancer center.

### Ethical Considerations

All participants provided written informed consent after receiving detailed information about the study’s purpose, procedures, potential risks and benefits, and their right to withdraw at any time without penalty, ensuring ethical adherence and voluntary participation. Participants provided informed consent electronically as part of the survey screening process. Additional verbal consent was obtained from all participants before commencing audio recording during the interviews. This study received approval from the institutional review board (STU00217509) at Northwestern University before participant recruitment and data collection. To protect participant privacy and confidentiality, all interviews were conducted in private settings, and audio recordings were securely stored on university servers accessible only to authorized research team members. Participants were assigned unique identification codes, and all identifying information, including names and contact details, was removed during data transcription. As compensation for their participation and time, individuals who completed the interview process received US $50 via a stored value electronic gift card (Hyperwallet). Finally, we adhered to established guidelines for qualitative research reporting [77] to maintain methodological transparency and rigor throughout the study (Checklist 1).

### Data Collection

#### Recruitment

The recruitment and data collection for this study took place between August and December 2023. Participants were recruited from clinics in a large National Cancer Institute–designated comprehensive cancer center located in Chicago, IL. Recruitment initially occurred through oncologist referral and digital advertisement in a survivorship clinic and a Spanish-speaking clinic to ensure inclusion of diverse patient populations. Subsequently, we expanded the recruitment channels to include the institution’s electronic health record patient portal, which became the primary recruitment channel. A message was disseminated to patients receiving cancer care through the patient portal inviting them to indicate interest in learning more about the study. Participants who expressed interest were then contacted via email with an introductory message and a link to complete the consent form and screening survey on REDCap (Vanderbilt University Medical Center). The survey served as a screening tool to identify participants eligible for follow-up interviews.

Eligibility for interview participation was determined based on predefined criteria. From the survey responses, participants were sampled for the semistructured interview if they (1) had a cancer history; (2) reported at least 2 health risk behaviors, defined as current smoking (every day or some days), BMI≥25 kg/m^2^, or engaging in less than 150 minutes of physical activity per week; (3) being 18 years or older and proficient in English; and (4) indicated interest in health behavior change (responding yes or maybe to intention items), meaning they were considering at least 2 of the following health risk behaviors: smoking, insufficient physical activity, and poor quality diet.

The survey responders who met the inclusion criteria and consented to be contacted for an interview were contacted via email and invited to participate in the semistructured interviews. A total of 16 participants were enrolled and completed the semistructured interviews. Participant recruitment continued until no new insights were observed. All interviews were completed and transcribed prior to analysis. Preliminary analysis informed code development, and coding proceeded until data saturation was reached [78], and we no longer identified additional novel information.

#### Study Instrument Design

Two senior researchers (Courtney Scherr and BS) led the design of a structured survey screener to collect demographic data, cancer history, current health behaviors, and participants’ attitudes toward behavior change. The survey included questions on motivation for change, perceived barriers, prior engagement with health promotion resources, and interest in participating in a digital health behavior change program.

The interview guide was collaboratively developed by the research team, incorporating input from oncologists, behavioral scientists, and patient advocates. The team had up to 4 meetings to collaboratively develop and iteratively refine the interview guide. The last meeting served as a pilot test where all the researchers who led the interviews tested the guide to establish familiarity, while other team members observed to assess clarity, relevance, and flow. Feedback from this informal pilot test informed minor refinements to question wording and sequencing prior to full data collection. The final interview guide consisted of 15 core questions covering key topics, including motivation for multiple health behavior change, perceived barriers to behavior change, cancer history and its influence on health behaviors, experiences with existing health promotion resources, and preference for ongoing support during intervention. See in Multimedia Appendix 1 for the interview guide, which contains the interview questions.

#### Semistructured Interviews

Interviews were conducted via the Zoom (Zoom Video Communications) videoconferencing platform, allowing for real-time verbal and nonverbal cue analysis. Each interview lasted between 30 minutes and 1 hour and was recorded. Two researchers were present during each interview: one acted as the primary interviewer, while the second took detailed notes (memos) to capture contextual nuances. Interviews followed a semistructured format, allowing flexibility for interviewers to probe and participants to elaborate on their experiences [79-81]. Participant recruitment continued until we reached data saturation, with no additional novel information emerging from subsequent interviews [78].

### Data Analysis

Survey responses were downloaded from REDCap and analyzed using R (R Foundation for Statistical Computing). We conducted descriptive statistical analyses to summarize participant characteristics, prevalence of health risk behaviors (smoking, diet, and physical activity), and combinations of co-occurring risk factors.

We further examined participants’ preferences for telehealth modalities and timing of intervention engagement using frequency distributions and cross-tabulations. Branching logic embedded in the survey was accounted for during analysis to ensure that only eligible responses were included in relevant items. The descriptive findings informed the purposive sampling strategy for interview recruitment.

All interviews were audio-recorded and automatically transcribed via Zoom. Before analysis, transcripts were reviewed and manually corrected for accuracy. During this process, the research team also ensured that all transcripts were verbatim and anonymized to remove participants’ identifiable data.

To analyze the data, we followed an abductive approach to thematic analysis [75,76], combining inductive data-driven coding with theoretical refinement. In the initial phase, we performed inductive thematic analysis to generate codes and identify preliminary themes that emerged organically from the data itself. Based upon the theory’s fit with observed emerging themes from the first round, the analysis then used self-determination theory (SDT) [64,69,82] as a theoretical interpretation framework to refine the themes and categorize the support needs and preferences of survivors of cancer for engaging in digital health behavior change interventions. SDT [64,69,82] is a well-established framework for understanding motivation, as it offers to explain the reasons—why and how individuals engage in a specific behavior [82]. It identifies the psychological elements and mechanisms that influence motivated behaviors [82], highlighting 2 primary forms of motivation: autonomous and controlled [83]. Interventions grounded in this theory have proven effective in fostering adoption (initial uptake) and facilitating actual engagement (active participation) in health behaviors among diverse groups, settings, and behavioral activities [61,63,84-86].

In total, 6 researchers were involved in the data analysis process. All 6 researchers initially familiarized themselves with 3 transcripts, noting preliminary observations and patterns. Of the 6, a total of 3 researchers conducted open coding to identify initial themes and patterns, while 3 researchers supervised the process and led the team discussions to ensure analytical rigor. Following independent coding sessions, the 6 researchers convened to examine emerging patterns and develop a preliminary codebook through consensus-based discussions [87]. The team used an iterative approach, with the 3 coders independently analyzing additional transcripts. Thereafter, the research team reconvened to discuss findings, resolve discrepancies, and refine the codebook accordingly. Through a recursive process, cumulatively involving 8 weekly 1-hour team discussions, we identified and defined final themes, returning to original codes when necessary.

## Results

### Participant Data—Demographics, Health Behavior, and Cancer-Related Characteristics

A total of 16 survivors of cancer participated in the interview study. Using December 2023 as a reference, participants ranged in age from 27 to 75 years (mean 47.7, SD 16.6 years). Most participants (13/16, 81.3%) were assigned female at birth, with 18.8% (3/16) assigned male at birth. All participants reported their current gender identity as consistent with their sex assigned at birth. The sample was predominantly White (10/16, 62.5%), with representation from Asian (2/16, 12.5%), Black or African American (2/16, 12.5%), Hispanic or Latino (1/16, 6.3%), and a participant who preferred not to disclose their racial identity (1/16, 6.3%). Over half of the participants (9/16, 56.3%) were employed full-time, and half of the participants (8/16, 50%) were married. In addition, most (9/16, 56.3%) had advanced degrees (MA, MS, MBA, PhD, MD, and JD), and all the participants were familiar with technology platforms. See Table 1 for detailed participant demographics and socioeconomic characteristics.

The time since cancer diagnosis, as reported by the participants, ranged from 1.1 to 21.7 years (mean 8.2, SD 6.6 years). All participants had elevated BMI levels, with a mean BMI of 34.4 (SD 7.3) kg/m^2^. Additionally, 7 of 16 participants reported 0 moderate-intensity exercise, and 10 of 16 participants did not meet recommended physical activity guidelines (0‐2 days per week), representing a critical need for health behavior change intervention. See Table 2 for details about participants’ health behavior indicators and cancer-related characteristics.

**Table 1.** Summary of study participants’ demographic data.

Characteristic	Values, n (%)
Age group (years)	
25-34	4 (25)
35-44	4 (25)
45-54	2 (12.5)
55-64	2 (12.5)
65-74	3 (18.8)
75+	1 (6.3)
Sex assigned at birth	
Female	13 (81.3)
Male	3 (18.8)
Race or ethnicity	
Asian	2 (12.5)
Black or African American	2 (12.5)
Hispanic or Latino	1 (6.3)
White	10 (62.5)
Other (preferred not to say)	1 (6.3)
Employment status	
Full-time employed	9 (56.3)
Retired	4 (25)
On disability	3 (18.8)
Marital status	
Married	8 (50)
Single or never married	4 (25)
Divorced or separated	3 (18.8)
Widowed	1 (6.3)
Highest level of education	
Advanced degree (MA, MS, MBA, PhD, MD, JD)	9 (56.3)
Some college or technical degree or associate degree	3 (18.8)
College degree (BA or BS)	2 (12.5)
High school graduate or GED^a^	2 (12.5)
Technology use	
Smartphone	16 (100)
Desktop or laptop or tablet computer	15 (93.8)
Wearable fitness tracker	6 (37.5)

a GED: General Education Development.

**Table 2.** Summary of participants’ health behavior indicators and cancer-related characteristics.

	Values
Cancer type, n (%)	
Breast	8 (50)
Leukemia	2 (12.5)
Colon and rectal cancer	1 (6.3)
Non-Hodgkin lymphoma	1 (6.3)
Thyroid	1 (6.3)
Hodgkin lymphoma	1 (6.3)
Multiple myeloma	1 (6.3)
Other: tongue, breast, thyroid	1 (6.3)
Time since most recent diagnosis (years)	
Mean (SD)	6.41 (5.35)
Range	2.18‐23.01
Time since last treatment (years)	
Mean (SD)	4.81 (6.00)
Range	0.06‐20.46
Health behavior indicators: BMI (kg/m^2^)	
Mean (SD)	34.4 (7.3)
Range	26.2‐50.2
Within past 6 months, doctor advised to lose weight, n (%)	
Yes	10 (62.5)
No	6 (37.5)
Moderate physical activity, n (%)	
0 None	7 (43.8)
3 days per week	5 (31.3)
2 days per week	2 (12.5)
1 day per week	1 (6.3)
5 days per week	1 (6.3)

### Thematic Analysis

#### Overview

This section presents an in-depth exploration of the findings from our thematic analysis across 4 major themes and 12 subthemes to directly address our research questions. In discussing what has worked well in past experiences and their desires in future interventions, we categorized participants’ preferences mapped onto 3 psychological needs from SDT: competence support through patient empowerment, technology-enabled tracking, and expert accessibility; autonomy support through personalized, flexible program design that acknowledges existing knowledge and provides choice; and relatedness support through human-centered systems featuring health promotionists, peer support, and health care team integration.

Although not classified as a primary psychological need, a fourth theme emerged that highlights participants’ desire for integrated, comprehensive care systems that connect behavior change programs with broader health care ecosystems rather than functioning as isolated programs. Textbox 1 highlights the key themes and the subthemes of this study.

Textbox 1. **Main thematic themes and subthemes that address participants’ support needs and preferences for facilitating autonomous motivation in digital behavior change interventions.**

**Theme 1: competence support through patient empowerment**

- Subtheme 1.1: ongoing recognition and positive reinforcement
- Subtheme 1.2: technology as a motivation enabler
- Subtheme 1.3: evidence-based health education

**Theme 2: autonomy support through personalized and flexible program design**

- Subtheme 2.1: guided autonomy and flexibility
- Subtheme 2.2: customization and sense of choice
- Subtheme 2.3: virtual access options

**Theme 3: relatedness support through human-centered systems**

- Subtheme 3.1: health promotionist as a central figure
- Subtheme 3.2: peer support with careful moderation
- Subtheme 3.3: empathy-centered communication style
- Subtheme 3.4: variable preferences for family involvement

**Theme 4: desire for integrated and comprehensive care systems**

- Subtheme 4.1: desire for whole-person care through complementary therapies
- Subtheme 4.2: health care team integration and data sharing

#### Theme 1: Competence Support Through Patient Empowerment

Participants consistently emphasized wanting programs that would build their sense of capability and effectiveness through ongoing recognition and reinforcement, comprehensive patient health education on lifestyle management, and technological enablement. These preferences reflected their desire to become more adept at managing their health rather than being passive recipients of care.

##### Subtheme 1.1: Ongoing Recognition and Positive Reinforcement

Ongoing recognition and positive reinforcement from health care providers emerged as a powerful mechanism for building competence and sustaining motivation. Participants valued it when providers acknowledged their efforts and achievements. Participant 15 recalled such a memorable experience, saying that:

*When [participant’s doctor] told me that he was proud of me, that I completed that medication trial ... I felt like I won the gold prize there.*

This recognition from trusted health care professionals validated the participant’s capabilities and reinforced their identity as an active, successful participant in their own care. The same participant expanded on how the recognition was relevant to their survivorship journey more broadly and implied their desire to receive such support in future interventions. They noted that:

I think that for any cancer patient, any encouragement and recognition that we can get for doing good things is great. Who doesn’t like recognition?

[Participant 15]

The emotional impact of such recognition and positive reinforcement suggests that, in addition to skill development, competence-building requires ongoing reinforcement and encouragement from respected sources, particularly the oncology care team. Oncologists and other cancer care team members, despite often being disconnected from health behavior change interventions, represent critical figures to whom survivors of cancer look for reinforcement and competence-affirmation.

While many participants described moments of encouragement that supported their sense of competence, a few expressed indifference about receiving behavioral change recommendations. For example, Participant 6 reflecting on repeated failed behavior change attempts highlighted a lack of confidence in achieving lasting outcomes despite exposure to programs or advice from their care team, sharing that:

So personally, it doesn’t always work for me. I hear the advice, but I don’t always apply the advice ... Just because I’ve done things like this, and it doesn’t always stick.

Participant 6 further mentioned that their inability to maintain behavior change was often reinforced by the absence of immediate or visible results, which made it difficult to assess progress: “I’m a very skeptical person, and so, if I can’t see applicable changes like right away, I don’t always apply the advice.” If Participant 6 could not see immediate results, they would be less likely to follow through with recommendations. These accounts suggest that in addition to skill development, competence is also shaped by continuous reinforcement in the form of meaningful feedback and tangible progress indicators.

##### Subtheme 1.2: Technology as a Motivation Enabler

Technology-enabled self-monitoring emerged as a crucial component for building competence by transforming abstract health goals into concrete, measurable actions. Participants shared past experiences with how technology served multiple accountability-enhancing functions, including providing objective self-tracking capabilities, facilitating professional monitoring relationships, supporting goal setting, and offering immediate behavior change feedback.

Participants reported that they found digital self-tracking and monitoring helpful when they participated in health behavior change. As one participant explained:

Just a month ago ... I was in the pretrial study. For two weeks, I had to record, using an app, ... to record what I eat, for me, it’s very helpful because it helps me see, okay, my protein goal is here, and fat goal is here.

[Participant 3]

Similarly, another participant highlighted how technology not only makes behavior change visible and measurable but also adds a social accountability component that facilitates behavior change, saying that “Well, I do the Apple rings, ... and I compete with my family member” (Participant 7). Using the Apple smartwatch rings encouraged the participant to complete daily movement, exercise, and standing goals. By competing with a family member, the participant engaged with the tracking features and also gained motivation from social interaction and friendly competition. This created a rewarding feedback loop, where closing rings and outperforming others reinforced healthy behavior.

Receiving immediate feedback proved particularly valuable in transforming their understanding of behavioral choices like food choices and dietary patterns. One participant described how technology reframed their eating habits, sharing that:

So that’s another reason that I find the recording app to be very helpful, because if I’m not thinking, because growing up I could just eat like a bowl of noodles with some soy sauce ... But then if you think about the nutrition content, there’s no protein, there’s no good stuff.

[Participant 3]

This immediate feedback helped the participants develop nutritional literacy by connecting familiar foods with their nutritional profiles, enabling more informed choices based on understanding rather than habit or assumption. Rather than seeing meals in cultural or habitual terms*,* the technology reframed these choices in nutritional terms, highlighting deficiencies that might otherwise remain invisible.

Participants also noted the role of technology in enhancing accountability with health care providers. For example, in Participant 11’s experience with diabetes management, the participant used wireless glucose monitoring and shared how the technology fostered transparency and accountability with their diabetes coach, enabling the coaching or support they received. Participant 11 noted that:

My meter was wireless, so she could see my readings ... I know she could see the numbers, so I couldn’t lie about it (laughter).

These findings suggest that interventions incorporating technological tools with appropriate human coaching elements might be particularly effective in creating the accountability structures that many survivors of cancer find motivating for health behavior change. Moreover, as behavior change programs become more common, prior experiences with digital self-management tools are clearly informing motivation in future programs.

##### Subtheme 1.3: Evidence-Based Health Education

Our study identified that evidence-based health information was particularly important to participants, who wanted to understand the rationale behind health promotion recommendations. They preferred receiving information from reliable, trustworthy sources rather than searching independently on the internet.

*I think that if somebody goes to the Internet, you don’t know what they’re gonna find. So having it all in one place, and knowing that it’s coming from a reliable source, I think, is a huge, trustworthy source as well*.

[Participant 5]

This preference reflected participants’ desire to build their competence on solid scientific foundations while avoiding the confusion and misinformation that can accompany independent internet searches. This insight suggests that framing health behaviors in terms of immediate health and wellness gains in addition to long-term outcomes like cancer recurrence prevention might enhance motivation for some survivors.

### Theme 2: Autonomy Support Through Personalized and Flexible Program Design

#### Overview

In addition to challenges related to competence, several participants described difficulties aligning recommended behavior change strategies with their personal routines and lived contexts. Participants often felt that the guidance they received was too generalized and did not account for the complexities of their daily lives. For example, one participant explained that although they were given behavioral change coaching, the recommendations did not reflect their individual circumstances and made implementation difficult.

*Sometimes when you get a whole plethora of information dumped on you ... Someone’s giving me a lot of advice and it’s all great advice bit it feels impossible to apply this advice, because the person doesn’t have any idea what my routine actually is ... it’s really hard sometimes to apply that to your routine. So that’s where I think I usually get caught up, is not being able to integrate it.*

[Participant 6]

This mismatch created a sense that behavior change strategies were externally imposed rather than self-directed, making them difficult to integrate and sustain. As a result, participants discussed that even well-intentioned recommendations were perceived as impractical or overwhelming, limiting participants’ ability to take ownership of their behavior change efforts. Accordingly, our study participants consistently emphasized wanting programs that could adapt to their individual circumstances, knowledge levels, and life constraints rather than requiring rigid adherence to standardized protocols. This preference for autonomy-supportive design reflected their desire to maintain control over their health journey while accommodating the complex realities of survivorship.

#### Subtheme 2.1: Guided Autonomy and Flexibility

Guided autonomy and flexibility emerged as fundamental requirements, as participants rejected programs with inflexible scheduling or mandatory time commitments. Several participants emphasized that rigid time commitments and intervention plans represented significant barriers to program engagement. As the conversations unfolded, participants shared their preference for collaborative programs that could adapt to their complex lives rather than demanding rigid adherence. One participant outlining their preference for exercise programs highlighted that:

So I think like whether it’s spaced out over different days in the week, or it starts off with like a little bit of time and then you can figure out where in my day what I protect this time. You know, if I’m building up to something, where could I kind of carve this out in terms of intensity?

[Participant 2]

Like Participant 2, most participants preferred the weekly distribution of activities over daily requirements. They advocated for starting small with gradual build-up, recognizing behavior change as a journey requiring low-barrier entry points. The participant also framed time allocation as personal exploration rather than an external mandate imposed by the program. The concept of “protected time” suggested that participants wanted programs to help them identify sustainable time allocation strategies. Intervention programs could help participants identify these protected times. Once identified, health and wellness time might become “protected time” that survivors defend against competing life demands, which could facilitate adherence.

Similarly, the importance of guided autonomy and schedule flexibility became reinforced when participants highlighted their desire for flexible re-engagement and how they would prefer the opportunity to pause, resume, and re-enter their behavior change programs, noting that:

There will probably be people who are interested who [...], have to miss a session, or have to reschedule stuff ... I guess that would just be something to consider like how to allow for people if they need to drop off for a bit and maybe get back on if that would be possible. Especially, I’m thinking right now, like I’m pregnant. So, if I had been in the program, probably have to modify my participation somehow, while I was pregnant, and then immediately postpartum like would not be doing any exercise, and my nutritional needs would be different.

[Participant 9]

The preference for pause-and-resume features reflected the reality that survivors of cancer often navigate multiple health challenges and life changes simultaneously.

Put together, participants showed a preference for programs that offer low barrier entry, gradual progression, flexible scheduling across the week, varied intensity-duration levels for activity scheduling based on individual capacity, flexible reengagement options, and, most importantly, support with identifying what schedule works for each patient.

#### Subtheme 2.2: Customization and Sense of Choice

Autonomy through customization and fostering survivors’ sense of choice emerged as another important preference for some participants. Among the challenges participants reported was the assumption that all participants started from the same knowledge baseline. We found that survivors sought recognition as informed patients with valuable prior learning. They want programs that assess and respect existing knowledge rather than enforcing universal curricula, thereby supporting their need for autonomy. Survivors wanted programs that acknowledged their existing health knowledge and expertise and tailored content based on their experience level. As one participant explained about a workplace weight loss program:

What felt challenging about it was they had like a lot of pre-programmed content about like these are the things you can focus on this week, and the content was like long and you couldn’t skip any of it ... It didn’t account for like the fact that you might already have some knowledge in certain areas.

[Participant 2]

The inability to skip known material transforms education from empowerment to obstacle, wasting time and engagement. This rigid structure and preset learning modules ignored the possible heterogeneous knowledge base among survivors of cancer and their varying educational needs and simply assumed uniform learning needs and pace. This feedback further underscored the need for adaptive interventions that could meet survivors at their current knowledge level.

In addition to acknowledging existing health knowledge, participants further highlighted their preference for choice and personal agency in intervention components. They wanted options for different activity types, duration commitments, dietary intake, and support levels rather than one-size-fits-all approaches. One participant, discussing their preference for choice on the intervention component they would like to receive, emphasized that:

*I think everybody’s unique. It’s not like one size fits all. When you have different diagnosis, it’s gonna be important to know where they fall short ... So I would just like, you know, if you have selections of things like you could check on here. This is what I’d like to receive information on: balance, strengthening.*

[Participant 11]

This preference reflected participants’ understanding that survivors of cancer have diverse needs based on their diagnosis, treatment history, health status, and information knowledge. While participants articulated their preference for choice, they also communicated their desire for support from the intervention in making the best choices, denoting an overall interest in shared or negotiated autonomy.

Although not explicitly articulated, our analysis throughout participants’ narratives implicitly depicts that interventions should be designed to avoid controlling language, as participants consistently described preferences for collaborative rather than controlled approaches.

#### Subtheme 2.3: Virtual Access Options

The convenience of virtual participation emerged as important for reducing barriers, particularly for those geographically distant from treatment centers. As one participant narrating their present ordeal with relocation and commute time noted that:

I think the telehealth piece is really helpful ... I know we talked a lot about time pressures today ... now that I live in a different area, it’s for some people, including myself, it’s like a bigger commute to get somewhere or do something in person.

[Participant 2]

Like Participant 2, most participants expressed the value of virtual options, not as a preference for digital over human interaction, but as a practical solution to address barriers, allowing survivors to access support regardless of their proximity to major medical centers.

### Theme 3: Relatedness Support Through Human-Centered Support Systems

Survivors of cancer consistently emphasized the irreplaceable value of human connection and support in health behavior change interventions, viewing technology as a useful tool but not a substitute for meaningful relationships with health care professionals, peers, and their care teams. This preference for human-centered approaches reflected their need for relatedness, including emotional support, shared understanding, and professional guidance that could adapt to their individual circumstances and challenges.

#### Subtheme 3.1: Health Promotionist as a Central Figure

We assessed participants’ receptiveness to health promotion support [88] by introducing the concept of a health promotionist, defined as a trained coach who helps individuals make health behavior changes. The responses were positive, with participants expressing interest in receiving this type of support and willingness to share behavior change data with their health promotionist.

Participants highly valued having a dedicated health professional who could offer expertise and hold them accountable in a personalized manner. One participant captured this sentiment, stating:

So I think like having a touch point that’s like a human being, whether it’s just for the fact that that’s interactive and motivating, or that’s like, this is the expert, right? Like this is a person who has like working knowledge in this area. I think that’s like very powerful.

[Participant 2]

This highlights a clear desire among participants for access to knowledgeable professionals who can provide tailored guidance instead of generic information or automated responses.

In addition to expert information, Participant 2 further highlighted the relevance of the supportive accountability aspect of health promotionist relationships. They shared that their past experience with human accountability significantly boosted their motivation compared to self-directed efforts. They recalled:

And that’s like, the most motivated I’ve ever been to exercise because there was, like, a person on the other end. So, if I, like, cancelled or didn’t go to the gym, like there was a human being who, like, was impacted directly by that.

[Participant 2]

The participant mentioned that their physical fitness trainer would check on them whenever they missed an appointment. This relational accountability fostered a sense of mutual responsibility that the participant found highly beneficial. The human element introduced reciprocal dynamics that enhanced their commitment to health behaviors.

When asked about their preferred way of receiving support from a health promotionist, participants emphasized a preference for human-centered support that enhances relatedness through understanding, acceptance, and practical guidance rather than judgment or control. Describing this supportive approach and relationship they would value, participants emphasized their desire for nonjudgmental support and guidance through setbacks and challenges. One survivor, while describing an ideal interaction, emphasized their interest in receiving supportive responses to difficulties and mentioned that:

Yeah, with respect, if I say, I had a Burger King Whopper today. “There’s no judgment there, you know, it’s okay. Well, you know, you can't do that, you know. But it’s okay” ... And when the mistakes happen, just help walk through that.

[Participant 11]

Participant 11 wanted the freedom to acknowledge their mistakes without the fear of harsh judgment. They appreciated the idea of a health promotionist recognizing that while their behavior might not be ideal, it can be addressed with understanding. This preference reflected participants’ understanding that sustainable behavior change involves navigating challenges and making mistakes, which requires supportive professionals who can help them and maintain motivation in the face of setbacks.

#### Subtheme 3.2: Peer Support With Careful Moderation

In addition to support from skilled health coaches, participants discussed their interest in receiving support from their peers. Participants reported finding profound value in connecting with other survivors who understood or could relate to their unique challenges and could provide emotional support through shared experience. One participant described the relief of shared experience:

So, another survivor that I know, we were just talking about weight gain this weekend. And she’s like my body is just different. I just don’t know what’s happening ... And so hearing that similar experience between the 2 of us, it is super reassuring to know that what’s happening to you isn’t only happening to you. It’s a common thing that happens to a bunch of people.

[Participant 6]

This validation that Participant 6’s and their friend’s struggles were common rather than personal failures provided emotional support that professional guidance alone may not replicate.

However, while participants recognized value in connecting with other survivors, they emphasized the critical need for skilled facilitation when peer support is offered in group settings to maintain supportive rather than detrimental dynamics. One survivor cautioned about potential unmoderated group challenges, noting that:

I guess I’m thinking about if you end up in a group with a lot of people, you know, you might end up with somebody who wants to hog all the attention all the time instead of just being there for the fun of, you know, wanting to drag you down with their story.

[Participant 7]

Consequently, the participant proposed that interventions should incorporate skilled moderation that could manage group dynamics effectively, saying that: “So, it’s so important for the leader to know how to manage that and to bring it back to a center point for everybody in the group so that you can have fun together” (Participant 7)*.* This preference highlighted participants’ understanding that peer support required structure and guidance to be beneficial rather than harmful.

#### Subtheme 3.3: Empathy-Centered Communication Style

Another suggestion that participants raised was empathy-centered communication that considers the struggles of cancer survivorship. Most participants preferred empathy and person-centered communication over clinical language. They emphasized the importance of using accessible language when health care professionals communicate with patients. For example, Participant 7, who reported experiencing information overload on receiving a 200-page book at diagnosis, while reflecting on the experience, highlighted the relevance and desire for human-to-human conversations. They mentioned that:

I remember they gave me this book that was like 200 pages long, I think, and it was overwhelming ... So, I think it’s great to have. It would be great to have someone speak to you, not necessarily in clinical terms. I think if, you know, instead of the clinical or medical side, speaking to you as a person would be really helpful.

[Participant 7]

Participant 7 described feeling overwhelmed by the volume of information. The issue was not necessarily the information itself but how it was communicated; specifically, the absence of relational, human-centered communication to contextualize complex information. Participant 7 valued having the same medically accurate and helpful information but delivered through personal, empathetic interaction rather than clinical documentation.

Similarly, another participant recounted a negative and judgmental experience with an oncologist who, according to the participant, strongly emphasized weight management but did so in a manner perceived as dismissive and unhelpful, sharing that:

I’ve had an oncologist before who I literally met twice, and he was very much a proponent of having a healthy weight, but he was very toxic about it. I had a friend who also saw him, and she was going through some rough chemo where she was vomiting all the time, and he was like, “Why don’t you try taking a hike? ... If you just lose a little bit of weight, you’ll stop vomiting.” And that was toxic. I dropped him so fast.

[Participant 1]

Furthermore, in highlighting their preferred communication style, another participant recalled an early encounter with a diabetic educator whose framing of behavior change felt condescending. They mentioned that:

The first diabetic educator I met with was like, “It’s been fun to live your life how you want to and eat what you want until now. And now it is time to get serious.” And I was like, hmm, that’s not helpful ... these sorts of condescending or infantilizing framings ...

[Participant 13]

Put together, these participants’ accounts emphasize how communication styles perceived as clinical, judgmental, or condescending can create emotional resistance and shape patients’ willingness to engage in future behavior change discussions.

#### Subtheme 3.4: Variable Preferences for Family Involvement

An interesting tension emerged between participants’ autonomy and relatedness needs regarding family involvement, illustrating the complex interplay between psychological needs in intervention design. Participants expressed variable preferences for how they would want their family involvement in their participation, with some participants wanting family engagement, while others preferred keeping their health behavior change participation private. This complex interplay revealed how supporting one psychological need might potentially conflict with another, creating nuanced design challenges for behavior change programs. While some participants viewed family engagement as essential for creating supportive environments that enhanced their sense of connection, others prioritized personal autonomy and privacy in their health behavior change journey.

For example, Participant 7 highlighted the benefits of family involvement, noting the relevance of having their family members enrolled in exercise programs to bolster survivors’ motivation. They shared that:

So, my thing is that maybe there’d be a way to bring people together to get them around exercise and healthy eating ... I mean it’s all good to have a support group for cancer, but to have families that are involved as well, so that you have that support when you go home.

[Participant 7]

This participant valued family engagement as a source of connection and ongoing support that would extend beyond formal program boundaries. Conversely, another participant’s experience highlighted the autonomy dimension. Expressing discomfort with family awareness of their participation in past behavior change intervention, they noted that:

*I was a bit embarrassed to tell people I was doing these programs. So, I didn’t really say anything to my friends or family.*

[Participant 1]

For Participant 1, the embarrassment from disclosure and the desire to maintain privacy and personal agency over disclosure outweighed potential relational benefits.

This tension illustrates how supporting one psychological need, in this case, relatedness through family involvement, might potentially undermine another, autonomy through personal choice. Interventions must navigate this complexity by offering flexible approaches to family involvement that could accommodate different comfort levels and family dynamics while respecting participants’ autonomy in deciding their level of disclosure and family engagement.

### Theme 4: Desire for Integrated and Comprehensive Care Systems

#### Overview

While the previous subthemes focused on individual psychological needs, participants also expressed distinct preferences that operated at the program and health care system levels, emphasizing the importance of structural integration and comprehensive service coordination. This subtheme represents survivors’ narratives that effective behavior change interventions must function as coordinated components within broader health care ecosystems rather than isolated programs addressing individual psychological needs alone.

Survivors of cancer consistently expressed a desire for behavior change interventions that would seamlessly integrate with their existing health care infrastructure and provide access to comprehensive wellness services. Rather than viewing health behavior change as a stand-alone activity, participants envisioned programs that would function as coordinated components of their overall care ecosystem, connecting them to broader support networks and addressing their multifaceted health needs.

#### Subtheme 4.1: Desire for Whole-Person Care Through Complementary Therapies

The desire for whole-person care approaches emerged as participants emphasized that their health needs extended far beyond cancer-related concerns. They wanted programs that could address the full spectrum of their health challenges rather than focusing narrowly on cancer survivorship issues. One participant articulated this comprehensive need:

If you have things [services] where people can explain the other things in their life going on. I mean, yes, there’s cancer, but cancer is not having much impact on my life. It’s all the other ones that are sitting there.

[Participant 249]

This perspective reflected the reality that many survivors were managing multiple health conditions simultaneously and needed comprehensive care approaches that could address their complete health care needs.

To facilitate their need for whole-person care, participants discussed their interest in accessing complementary wellness services. In addition to physical therapy and nutrition counseling, they wanted connections to various healing modalities, including mental health support and mind-body practices like yoga that addressed the emotional challenges of survivorship. One survivor described their interest in this movement and stress relief practices, saying that:

... also, like maybe meditation, yoga, of course, I’m really into yoga. It’s been something I do for my back and it does help. So maybe things like that ... And I also know people that do have cancer, they do suffer with pain and chronic pain. Maybe something like with massage therapy.

[Participant 12]

This preference highlighted participants’ holistic understanding of health and wellness that extended beyond medical interventions to include therapies that could address pain, stress, and overall quality of life.

#### Subtheme 4.2: Health Care Team Integration and Data Sharing

As part of measures to enhance holistic and comprehensive care delivery, participants shared their desire for continuity and coordination between their behavior change efforts and their ongoing medical care. Survivors wanted their clinical teams to be aware of and involved in their wellness activities to optimize care coordination. Participants also valued health care providers who took holistic approaches to their care, addressing not just medical issues but also supporting their overall well-being. One survivor described their appreciation for comprehensive provider support:

*My doctor [...] He’s the best oncologist I’ve ever had, because he treats the mind, the body, and the soul. [...] And so, you know, they’ve really been helpful, you know, and just keeping me on track of all the other things outside of the cancer diagnosis.*

[Participant 11]

This desire for integrated, whole-person care suggested that participants wanted their behavior change efforts to be supported and reinforced by their trusted health care providers rather than existing in isolation.

Participants wanted their clinical teams to be informed about and involved in their wellness activities. One participant articulated their comfort with data sharing: “I think that them knowing what I am or I’m not working on between visits, I’d be fine to have them like integrated into that process” (Participant 2). This preference reflected participants’ views that behavior change efforts should complement rather than exist separately from their medical care, allowing for better care coordination and more informed clinical decision-making.

The preference for integrated and comprehensive care systems ultimately reflected participants’ understanding that sustainable health behavior change required coordinated support across multiple domains. They envisioned programs that could bridge the gaps between different aspects of their care, connect them to appropriate resources, and provide holistic support that addressed their physical, emotional, and practical needs as whole persons rather than just survivors of cancer.

These findings, ultimately, suggest that survivors of cancer seek more than improved health outcomes when participating in multiple behavior change interventions. They envision programs that respect their autonomy while providing robust support systems. Their preferred intervention considers time constraints by providing flexible, personalized, and convenient support while strengthening relationships with health promotionists and clinicians through coaching, accountability, and ongoing communication. They also emphasized that effective interventions should be integrated into routine clinical care and recognize that successful behavior change depends on addressing survivors’ physical, emotional, and social needs alongside their cancer care. Finally, survivors desire practical education delivered in an empathy-focused and accessible language structure that acknowledges both their accumulated health information and ongoing vulnerabilities in navigating life after cancer.

## Discussion

### Principal Findings

This qualitative study examined the pre-enrollment perspectives of survivors of cancer on digital health behavior change interventions, with particular focus on understanding their support preferences for sustaining long-term engagement. Applying the SDT [64,68,69,82] as a theoretical perspective to frame the participants’ data, 3 themes emerged on participants’ support preferences that address meeting the basic psychological needs of survivors of cancer for maintaining autonomy. First, participants wanted skill development and mastery support (competence) through technology-enabled tracking and access to evidence-based health education. Second, participants discussed their interest in personalized and flexible program design (autonomy) that acknowledged their existing knowledge, provided scheduling flexibility, and respected their individual circumstances. Third, the study uncovered participants’ desire for human-centered support systems to enhance relatedness through dedicated health promotionists [88], carefully facilitated peer support, and integrated with their clinical care teams. While most of our findings centered around the psychological needs of survivors of cancer, we identified participants’ need for integrated, systems-level support that connected behavior change interventions with the broader health care ecosystem of survivors of cancer rather than treating such programs as isolated interventions. Overall, this study demonstrates complementarity with and extends prior foundational work [89] that identified motivation and behavior change techniques for SDT-based health interventions through expert consensus. Where the expert-driven approach of Teixeira et al [89] identified what techniques should theoretically support psychological needs, our pre-enrollment study reveals how survivors of cancer want these techniques delivered and what specific program features would make them meaningful and sustainable.

### Comparison With Prior Work

#### The Central Role of Psychological Need Satisfaction

SDT [64,69,82] is a well-established framework for understanding motivation. Interventions grounded in this theory have proven effective in fostering adoption (initial uptake) and facilitating actual engagement (active participation) in health behaviors among diverse groups, settings, and behavioral activities [61,63,84-86,90]. It identifies the psychological elements and mechanisms that influence motivated behaviors [82], highlighting 2 primary forms of motivation: autonomous and controlled [83]. Changing autonomous motivation encourages individuals to engage in certain behaviors and sustain those behaviors over time, as reported in a meta-analysis review [91], which makes interventions grounded in SDT especially beneficial for health behavior change programs [92,93]. Based on this understanding and from the participants’ perspective, we identified opportunities to facilitate health behavior change and make survivors of cancer more autonomously motivated by designing interventions to align with their needs and preferences.

Our study revealed participants’ interests, support needs, and preferences that primarily mapped onto the 3 basic psychological needs: competence, autonomy, and relatedness, which, according to SDT, are vital psychological needs for promoting autonomous motivation. Autonomy involves feeling a sense of control and personal choice, which enhances motivation when individuals are empowered to make decisions about their health. Competence refers to a person’s capacity to handle challenges and successfully achieve health goals and objectives. It accounts for the necessary knowledge and skills to implement decisions. Relatedness pertains to establishing meaningful connections with others, which can enhance motivation by offering emotional support and fostering a sense of belonging. In the subsequent paragraphs, we discuss evidence-based recommendations for intervention design that specify the psychological mechanisms through which effective programs might operate.

#### Supporting Competence Through Patient Empowerment

Empowering patients to make informed decisions, advocate for their needs, and share decisions toward their health outcomes has been widely discussed in the literature [94-96], and its relevance has been highlighted as a motivation for action [97] toward better self-management of chronic disease [98-101]. Participants’ emphasis on evidence-based education, ongoing recognition and reinforcement, and technological enablement reflected their desire to become adept at managing their health rather than being passive recipients of care. When previously enrolled in health behavior change, participants saw the relevance of technological tools in enabling their participation by fostering accountability and transparency with the care team and improving patients’ health promotion literacy. They highlighted the value of technology in facilitating goal-setting, self-tracking, and monitoring, and receiving immediate feedback to enhance patient engagement.

Additionally, our study identified the desire of survivors of cancer for evidence-based health education and health literacy. Although operationally distinguished from patient empowerment, in that being health literate alone does not translate to an empowered patient [94,97], research posits that health literacy and empowerment work together to enhance self-management skills. Based on participants’ data, we argue that providing patients with the skills to make reasoned decisions and informed choices may facilitate their continued motivation to participate and engage with health promotion interventions. However, a critical implementation consideration for this finding is the need to empower patients with health education and literacy support, with careful attention to preserving survivors’ autonomy rather than reinforcing practitioner authority. While our study participants valued evidence-based information and expert support, empirical evidence demonstrates that seemingly supportive techniques can backfire if implemented in controlling ways. A meta-analysis reported that offering structure and information was associated with lower autonomous motivation, possibly because participants experienced these strategies as controlling or reinforcing the providers’ authority in guiding behavior change [86]. Our participants’ perspectives for implementing knowledge recognition systems that acknowledge survivors’ existing expertise, offering adaptive content, and ensuring expert accessibility through collaborative consultation could provide pathways for delivering essential information in autonomy-supportive ways.

The desire for recognition and positive reinforcement from health care providers further highlighted the social nature of competence building. Participants valued validation of their efforts from trusted professionals, suggesting that competence support requires patient empowerment through positive reinforcement and recognition from respected sources. Providing survivors of cancer with recognition and positive reinforcement can further improve their self-efficacy and empowerment toward adopting and maintaining healthier lifestyle choices.

#### Fostering Autonomy Through Personalization and Choice

Perhaps the strongest theme in our data was participants’ desire for autonomy expressed through a desire for shared decision-making and a preference for personalized, flexible program design that respected their individual circumstances and expertise. The survivors of cancer in our study expressed high interest in taking increased responsibilities and shared decision-making when they participate in health behavior change interventions as opposed to being passive recipients of care.

As part of their autonomy-supportive techniques, Teixeira et al [89] recommended identifying obstacles or sources of pressure for participants. Our study advances this contribution by specifying some concrete implementation requirements, including pause-and-resume features for life transitions and incremental physical activity intensity options to help participants self-manage challenges. Notably, corroborating Teixeira et al [89], we identified participants’ desire for personal agency and choice over intervention features vis-à-vis customization of intervention content. For example, participants wanted programs that would allow them to skip familiar content and build on existing expertise. We found that many survivors of cancer accumulate substantial health knowledge through their active treatment and are somewhat aware of behavior change requirements for survivorship. Programs should acknowledge this awareness to prevent inadvertently undermining autonomy by treating participants as universally lacking health literacy.

While autonomy has been identified as a major human need to facilitate internalization of health behaviors [67,68] and that choice is a positive motivator [102], it has also been argued that having many opportunities for choices might in fact be burdensome and demotivating [103] or generally energy-draining, thereby complicating decision-making. Our study echoes our participants’ vantage on their desire to exercise their capacity to reflectively endorse or reject certain intervention features, which is the SDT’s view of autonomy [89,104]. SDT endorses facilitating people’s experience of choicefulness or volition, which may happen with one, more, or fewer options, as the number of options is not, by itself, defining autonomy [69]. A person might have multiple choices but still lack a sense of autonomy, feeling instead burdened and frustrated by the decision-making process. On the other hand, an individual could find themselves with just a single choice, essentially having no options, yet still feel a sense of autonomy if they genuinely support that choice.

Moreover, our findings reveal that autonomy can be guided or negotiated, thereby giving intervention designers the flexibility to remain objective in intervention research design. We see incorporating frequent patient-centered interactions, in other words, check-ins, throughout the intervention lifecycle as a potential pathway for guiding or negotiating autonomy.

Although not explicitly articulated, our reflective analysis throughout participants’ narratives implicitly depicts that interventions should be designed to avoid controlling language, as participants consistently described preferences for collaborative rather than controlled approaches. This finding aligns with recent empirical evidence on SDT intervention mechanisms [86]. The meta-analysis demonstrated that theoretically predicted techniques like noncontrolling language and providing rationales significantly enhanced autonomy satisfaction and autonomous motivation, respectively.

#### Enhancing Relatedness Through Human-Centered Support Systems

In addition to needs and preferences that satisfy competence and autonomy, our study identified the support needs of survivors of cancer for relatedness through human-centered support. Relatedness accounts for people’s desire to feel accepted and close to others, as opposed to feeling isolated or rejected [64,65,69], and it reinforces motivation by linking health behaviors to one’s community. Our study participants consistently emphasized the value of human connection and support. The desire for health promotionists as central figures in care for survivors of cancer reflected a need for expert support and professional guidance, but, more importantly, meaningful relationships that could provide accountability and emotional support through the challenges of behavior change.

Additionally, while participants valued receiving support from dedicated health promotionists, they also noted their interest in receiving support from their peers. The survivors of cancer in our study discussed the opportunity to receive relational and emotional support from connecting with other survivors, for example, in group settings, who understood their unique situations and with whom they shared similar experiences. However, our participants highlighted the need for skilled moderation when peer support is offered in group settings to maintain supportive rather than detrimental dynamics. Moreover, a recent meta-analysis showed that group-based intervention activities demonstrated dual effects, in that participants reported increased feelings of connection and belonging during group interactions, yet experienced diminished individual competence perceptions when functioning independently [86]. This suggests that intervention designers should pay careful consideration to delivering group support to prevent inadvertently undermining personal self-efficacy.

An interesting tension emerged between participants’ autonomy and relatedness needs. Participants expressed variable preferences for how they would want their family involved in their participation, with some participants wanting family engagement, while others preferred keeping their health behavior change participation private. Interventions must navigate this complexity by offering flexible approaches to family involvement while respecting participants’ autonomy in deciding their level of disclosure and family engagement.

While Teixeira et al [89] identified adopting empathetic listening as a relatedness-supporting technique, our survivors articulated the specific qualities they want in health promotionist relationships, including incorporating nonjudgmental acceptance, practical guidance through setbacks, an empathy-centered communication style, and collaborative rather than controlling approaches. Paradoxically, an empirical study showed that personal engagement from practitioners, including showing individual interest and offering affirmations, was associated with reduced autonomy and relatedness satisfaction among participants [86]. The study suggested that this detrimental effect may occur because such approaches create participant dependence on the practitioner, or the results could reflect the heterogeneous effects of multiple techniques reviewed under the umbrella study. This study highlights survivors’ nuanced preferences for health promotionist relationships that balance support with autonomy, emphasizing collaboration rather than controlling involvement.

To facilitate relatedness, our participants consented to and expressed value in having their relevant behavioral participation and progress data shared with their primary and oncology care teams. This preference challenges the common practice of delivering behavior change interventions in isolation from survivors’ primary and oncology clinical care, suggesting instead that integrated approaches may more effectively support sustained engagement by leveraging existing trusted relationships and ensuring care continuity.

#### Systems-Level Integration and Comprehensive Care

Beyond individual psychological needs, participants expressed distinct interest in support needs that operated at the program and health care system levels. The emphasis on integration with existing care infrastructure and access to comprehensive wellness services reflects survivors’ holistic understanding of health and their desire for integrated rather than fragmented support.

This systems-level perspective has important implications for how digital health interventions are conceptualized and implemented. Rather than functioning as standalone programs, effective interventions may need to serve as coordinated components within broader health care ecosystems, connecting participants to complementary resources and facilitating communication between different aspects of their care. Participants’ interest in complementary wellness services, including counseling, mental health support, and mind-body practices, suggests that survivors understand wellness as multidimensional and are seeking programs that can support their complete health and well-being rather than focusing solely on specific behaviors or cancer-related outcomes.

#### Positioning Findings Within Broader Behavior Change Frameworks and Emerging Approaches

Our findings may also be understood in relation to other behavior change frameworks beyond SDT. First, the importance participants placed on accountability, encouragement, self-monitoring, and confidence-building aligns with social cognitive theory (SCT), which emphasizes self-efficacy, behavioral capability, and social support as key drivers of behavior change. From an SCT perspective, features such as coaching, feedback, and selective peer interaction may function not only as sources of encouragement but also as mechanisms for observational learning, modeling, and accountability that reinforce self-efficacy [105-108]. Prior work in cancer survivorship has shown that SCT-based physical activity and diet interventions can improve behavior, particularly in physical activity–focused programs, although results are more mixed for multiple-behavior interventions. Second, participants’ emphasis on recurrence risk, long-term health, and the need for clear, credible information about why behavior change matters is consistent with the health belief model, particularly its constructs of perceived susceptibility, perceived benefits, and perceived barriers. Survivors’ preferences for low-burden, trustworthy, and symptom-aware support may also reflect efforts to reduce cognitive and physical barriers while responding to salient cues to action, such as symptom changes or fear of recurrence. Empirical studies show that perceived benefits and barrier self-efficacy are associated with greater engagement in moderate-to-vigorous physical activity among survivors of cancer [109,110], and that perceived barriers, such as fatigue, comorbidities, or treatment side effects, remain central determinants of behavior change [111,112]. Third, participants’ descriptions of hesitation, trial-and-error, and varying preferences for when and how to engage in behavior change echo the transtheoretical model’s focus on stages of readiness. Transtheoretical model–based health behavior change studies suggest that preferences for specific forms of support, such as informational guidance, reminders, or other accountability features, which were also identified in this study, may shift over time, as individuals progress through interventions [113,114]. Our study highlights the importance of matching support to an individual’s current level of preparedness. Future work may study the relevance of stage-sensitive design in identifying how various forms of digital support may feel most useful at different stages of patients’ health behavior change. Taken together, these comparisons suggest that the preferences identified in our study, framed through motivation and pre-enrollment preferences, also support broader behavior change principles.

In addition to studies that used other theories, we identified some preference-focused studies in cancer survivorship that suggest that survivors value features such as tailored feedback, coaching, and optional social support [115-117], which we also identified in this study. While these prior studies identify which features are desirable, our findings extend this work by including perspectives from survivors of more diverse cancer types and examining multiple health behaviors. More importantly, our study elucidates the underlying motivational pathways, contextual constraints, and emotional experiences that shape these preferences.

Finally, as AI becomes increasingly ubiquitous, it offers a promising approach for making digital behavior change interventions more responsive to the lived realities of survivors of cancer [118,119]. Our findings highlight survivors’ preference for personalized, holistic, and context-sensitive support. Emerging evidence in the literature suggests that AI-enabled digital behavior change interventions can effectively deliver such support through personalization [120-122], adaptive coaching [123,124], predictive risk modeling [125,126], and multimodal data integration [127]. For example, a randomized trial among sedentary survivors of cancer demonstrated that an AI-enabled, bidirectional voice coach produced meaningful short-term increases in physical activity (steps per day), supporting the feasibility of AI-driven adaptive coaching [123]. More broadly, context-aware interventions using analytic and AI methods to integrate multimodal data such as patient-reported outcomes, wearable-derived activity and sleep signals, and clinical context have been used to automate individualized feedback or monitoring and were associated with improvements in behaviors, including physical activity and diet [128]

While we see the promise of AI, our study participants’ desire for encouragement and human connection that does not feel purely automated highlights careful considerations for fully automated approaches. Hybrid programs that combine AI scalability with human relational support may be best positioned to sustain engagement and acceptability [129]. We recommend that, rather than viewing technology and human support as alternatives, effective programs should leverage technology to augment and enhance human-centered survivorship care.

#### Summary of Recommendations

Our findings provide specific, actionable recommendations for developing digital health behavior change interventions that can address the complex motivational and support needs identified by survivors of cancer while maintaining focus on sustainable, autonomous motivation and engagement (Textbox 2).

Textbox 2. **Summary of recommendations for designing digital health behavior change interventions for survivors of cancer.**

**Flexible engagement architecture**

- Build pause-and-resume functionality with easy re-entry pathways for life transitions
- Offer graduated intensity levels with low-barrier entry points (starting with 10‐15 minutes and building to longer sessions)
- Provide weekly activity distribution options rather than requiring daily participation
- Include knowledge assessment tools that allow users to skip familiar content and access advanced modules

**Supporting motivational progression**

- Frame immediate benefits (eg, energy, mood, and sleep) prominently alongside long-term health outcomes
- Use collaborative language rather than prescriptive or controlling directives
- In addition to long-term outcomes, connect health behaviors to personally valued outcomes (family time and quality of life)
- Include progress celebration and provider recognition features
- Create achievement systems that acknowledge effort and improvement, not just outcomes

**Technology and human integration: enhanced monitoring and feedback**

- Implement wireless goal tracking that provides real-time analysis and goal progress
- Create dashboards that make abstract health concepts concrete (dietary intake goals and activity patterns)
- Enable data sharing with designated health coaches and clinical teams
- Provide immediate feedback that transforms understanding of goal choices and activity impact

**Technology and human integration: human-centered support systems**

- Assign dedicated health promotionists trained in nonjudgmental, collaborative coaching approaches
- Train all support staff in autonomy-supportive communication techniques, including establishing clear protocols for supporting users through setbacks without judgment
- Include video-based check-ins (preferred over phone or text) with flexible scheduling
- Provide expert accessibility for situation-specific guidance (eg, travel, holidays, and setbacks)
- Offer both individual coaching and facilitated peer support groups with skilled moderators

**Health care integration: clinical care coordination**

- Develop automated summary reports for oncology teams before routine appointments
- Create shared care plans accessible to primary care, oncology, and wellness teams
- Establish referral pathways to complementary services such as nutrition, mental health, and physical therapy
- Flag cancer history prominently in electronic health records to trigger proactive screening

**Health care integration: comprehensive service access**

- Partner with existing clinical dietitians, mental health professionals, tobacco treatment specialists, and fitness specialists
- If resources permit, provide connections to mind-body services (yoga, meditation, and massage therapy)
- Address noncancer health challenges within the same platform
- Create service menus allowing users to select relevant support combinations

**Personalization and choice: adaptive content delivery**

- Implement branching logic based on prior health knowledge and experience level
- Offer multiple communication preferences (video, phone, text, and in-person options)
- Provide choice in family involvement levels with privacy controls
- Include realistic goal-setting that accommodates existing lifestyle patterns
- Allow modification of goals and approaches based on changing circumstances
- Avoid time-pressured or mandatory completion requirements

**Personalization and choice: individual preference recognition**

- Allow customization of activity types, nutritional approaches, and support frequency
- Respect varying comfort levels with technology through alternative access methods
- Accommodate different learning styles and information processing preferences
- Enable users to set their own success metrics beyond standard clinical outcomes
- Include decision-making tools that help users choose approaches aligned with their values

### Limitations and Future Research

This study was conducted with a predominantly English-speaking, well-resourced survivor of cancer sample from a single geographic region. Participants were highly educated, mostly women, and White, and reported high technology and health literacy skills. Moreover, recruitment through patient portals may have introduced selection bias by overrepresenting individuals who are more engaged in their health care or digitally connected. These factors may limit the generalizability of findings to more diverse survivor populations. However, it is worth noting that the recruitment approach aligns with our study’s focus on digital and telehealth-supported interventions. Additionally, this study included efforts to recruit Spanish-speaking survivors of cancer to enhance diversity.

While this study may be limited in its recruitment and sampling approaches, it highlights some key strengths of its contributions. We analyzed the data from an SDT perspective, a prominent human motivation theoretical framework with constructs that have been well-established as universal psychological needs. Hence, the findings may be relevant and applicable to other survivors of cancer. Additionally, the pre-enrollment perspective, while offering unique insights into anticipated needs and preferences, may not fully capture the reality of actual program participation. Our focus on the pre-enrollment period means we have not directly assessed how these preferences might evolve through actual program participation or how well they predict long-term engagement. Nonetheless, research has established that understanding and designing systems around user needs and preferences make systems more likely to be adopted and used consistently because they fit into users’ lives and routines [130,131].

Future research should examine whether these expressed support needs and preferences translate into engagement and outcomes when implemented in actual interventions. Additionally, longitudinal studies following survivors through the complete intervention experience would provide valuable insights into the dynamic nature of motivation and preference evolution over time. Specifically, given the rising adoption of AI in digital health, future work should explore holistic frameworks for leveraging AI to augment autonomous motivation and enhance patient engagement in interventions.

Finally, the SDT framework, while providing a useful structure for analysis, may not capture all relevant aspects of motivation in cancer survivorship. SDT’s focus on individual-level motivation underemphasizes structural and contextual barriers. Future research might explore how other theoretical frameworks complement or extend SDT’s insights, particularly regarding the unique psychological challenges and opportunities that cancer experience creates for behavior change motivation.

### Conclusions

This study demonstrates that survivors of cancer possess ample understanding of their motivational needs and can provide valuable guidance for intervention design when given the opportunity to articulate their preferences. Our research addresses a critical gap identified in recent literature that, despite the recognized importance of the perspectives of survivors of cancer, few digital health studies involve survivors in research design and development. By centering survivor voices in the pre-enrollment phase, this study provides concrete, actionable recommendations for developing digital health interventions that can facilitate survivors’ natural progression from externally motivated participation toward sustained, autonomous engagement with health behaviors.

This research provides a roadmap for developing digital health interventions to close the critical gap between evidence-based lifestyle recommendations and current survivor adherence patterns. By adopting the flexible, human-centered, and integrated approaches outlined in our findings, digital health interventions can better fulfill their promise of providing accessible, effective support for health behavior change in cancer survivorship.

## Supplementary material

10.2196/87514 Multimedia Appendix 1 Interview guide.

10.2196/87514 Checklist 1 21-Item SRQR checklist for reporting qualitative research.

## Abbreviations

SCT: social cognitive theory
SDT: self-determination theory
