# Supplementary material for: Designing for Autonomous Motivation: Qualitative Interview Study on Pre-Enrollment Preferences of Survivors of Cancer for Digital Health Behavior Change Programs
Source: J Med Internet Res. 2026 Aug 10;28:e87514. doi: 10.2196/87514 (PMC13455853; doi:10.2196/87514)
Supplement: Supplementary file 1 [file jmir-v28-e87514-s001.pdf]

STELLAR Pilot 1 Semi-Structured Interview Guide

Introductions

Hello, my name is ___________. I am (role in study). Thank you for agreeing to participate in an interview as part of the STELLAR research center. STELLAR is funded by the National Cancer Institute Cancer Moonshot Initiative. We appreciate your willingness to share your thoughts with us about Health Behaviors and use of Telehealth in cancer care.

Purpose

I/ The STELLAR Multiple Principal Investigators in the Department of Preventive Medicine and Medical Social Sciences at Northwestern University am/are interested in developing a cancer-specific telehealth program that will be fully integrated into cancer care to help cancer survivors live healthier lives following their cancer diagnosis.

The purpose of our session today is to learn more about your preferences, ideas, and opinions regarding the types of physical activity promotion, healthy weight management, and smoking cessation programming you think would be useful to integrate into cancer survivorship care via telehealth. Findings from today's session will help shape how we move forward in designing the program.

We are going to start off by going over the ground rules for today's session.

Guidelines

a. *There are no right or wrong answers. Please feel free to share your point of view. We are genuinely interested in your perspective.*
b. *Keep in mind that we're just as interested in negative comments as positive comments, and at times the negative comments are the most helpful.*
c. *We will record today's session because we don't want to miss any of your comments. People often say very helpful things in these discussions, and we can't write fast enough to get them all down. Are you okay with this?*
d. *We're on a first name basis. However, we will not use any names in our reports. That being said, if you do not want to use your real name because of the recording that is fine. Please let us know what you would like to be called.*
e. *Do you have any questions before we start?*

Cancer History

1. Can you please tell me a little bit about your cancer diagnosis, when you were diagnosed, and the treatment you received? (*Note to interviewer: this should not be lengthy, we are interested in context and opening the interview here*).
2. Since entering survivorship, have you felt more or less motivated to improve your health? (e.g., exercise, manage weight, quit smoking).

Health Behaviors

Physical Activity

**Ask the following questions if participant qualifies as having insufficient physical activity based on survey responses or eligibility screener:*

3. What does your physical activity look like in a typical week?
    a. If you have tried to make changes to your physical activity, what are some of the biggest barriers you have faced?
        i. Are there any particular challenges due to being a cancer survivor?
4. If there was a physical activity program for post active curative intent treatment cancer survivors who completed treatment (surgery, radiation, chemo) what would you like that to look like?

Weight Management

**Ask the following questions if participant qualifies as a weight loss candidate based on survey responses or eligibility screener:*

5. Do you follow a special diet for weight management /weight loss or for cancer control that you find helpful?
    a. [If yes] What kind of diet are you following?
    b. [If yes] How did you learn about it?
    c. [If no] What does your typical diet look like?
6. If you have tried to make changes to your diet, what are some of the biggest barriers that you have faced?
    a. Are there any particular challenges due to being a post active curative intent treatment cancer survivor?
7. If there was a weight management program for cancer survivors who completed treatment (surgery, radiation, chemo) what would you like that to look like?

Smoking

**Ask the following questions if participant qualifies as a smoking cessation candidate based on survey responses or eligibility screener:*

8. What does your usual daily or weekly smoking habit look like?
9. If you've tried to quit or cut down on your smoking what are the barriers you have faced?
    a. Are there any particular challenges due to being a post active curative intent treatment cancer survivor?
10. If there was a smoking cessation program for cancer survivors who completed treatment (surgery, radiation, chemo) what would you like that to look like?

<u>General Behaviors</u>

11. [ASK FOR RELEVANT BEHAVIORS] What are your thoughts about changing your physical activity, your calorie intake, and your smoking at the same time?
12. How do you think about these activities (smoking, weight management/loss, physical activity) as they relate to cancer risk or cancer recurrence?

<u>Health Promotionist</u>

13. What do you think about meeting with a health promotionist (a coach with extra training in helping people make health behavior changes) to help with becoming more active, managing weight, and/or quitting smoking?
    a. Would you be interested in sharing your tracking data with your health promotionist?
        a. [If no]: or why not?

<u>Care Team Engagement</u>

14. If you were to participate in a program that promotes weight management/loss, increased physical activity, and/or smoking cessation, would you want your cancer care team to be involved?
    a. <u>If yes how?</u>

<u>Closing Remarks</u>

15. Is there anything else that you think the study team should consider in trying to design a telehealth program for cancer-specific health behavior change?

Thank you so much for participating today. This information is helpful and will help us design a more effective intervention.

In the meantime, you should receive your e-giftcard in your e-mail within the next few weeks.

If you have any questions, or additional feedback on anything we have discussed today, please feel free to contact us using the information provided in the informed consent.
