# Supplementary material for: Designing for Autonomous Motivation: Qualitative Interview Study on Pre-Enrollment Preferences of Survivors of Cancer for Digital Health Behavior Change Programs
Source: J Med Internet Res. 2026 Aug 10;28:e87514. doi: 10.2196/87514 (PMC13455853; doi:10.2196/87514)
Supplement: Supplementary file 2 [file jmir-v28-e87514-s002.docx]

| **No** | **Topic** | **Item [Y = Included, N = Not Included]** |
| --- | --- | --- |
| **Title and Abstract** | | |
| S1 | Title | Y |
| S2 | Abstract | Y |
| **Introduction** | | |
| S3 | Problem formulation | Y |
| S4 | Purpose or research question | Y |
| **Methods** | | |
| S5 | Qualitative approach and research paradigm | Y |
| S6 | Researcher characteristics and research reflexivity | Y |
| S7 | Context | Y |
| S8 | Sampling Strategy | Y |
| S9 | Ethical issues pertaining to the human subjects | Y |
| S10 | Data collection methods | Y |
| S11 | Data collection instruments and technologies | Y |
| S12 | Units of study | Y |
| S13 | Data processing | Y |
| S14 | Data analysis | Y |
| S15 | Techniques to enhance trustworthiness | Y |
| **Results/Findings** | | |
| S16 | Synthesis and interpretation | Y |
| S17 | Links to empirical data | Y [available upon request] |
| **Discussion** | | |
| S18 | Integration with prior work, implications, transferability, and contributions(s) to the field. | Y |
| S19 | Limitations | Y |
| **Other** | | |
| S20 | Conflicts of interest | Y |
| S21 | Funding | Y |
|  |  |  |
